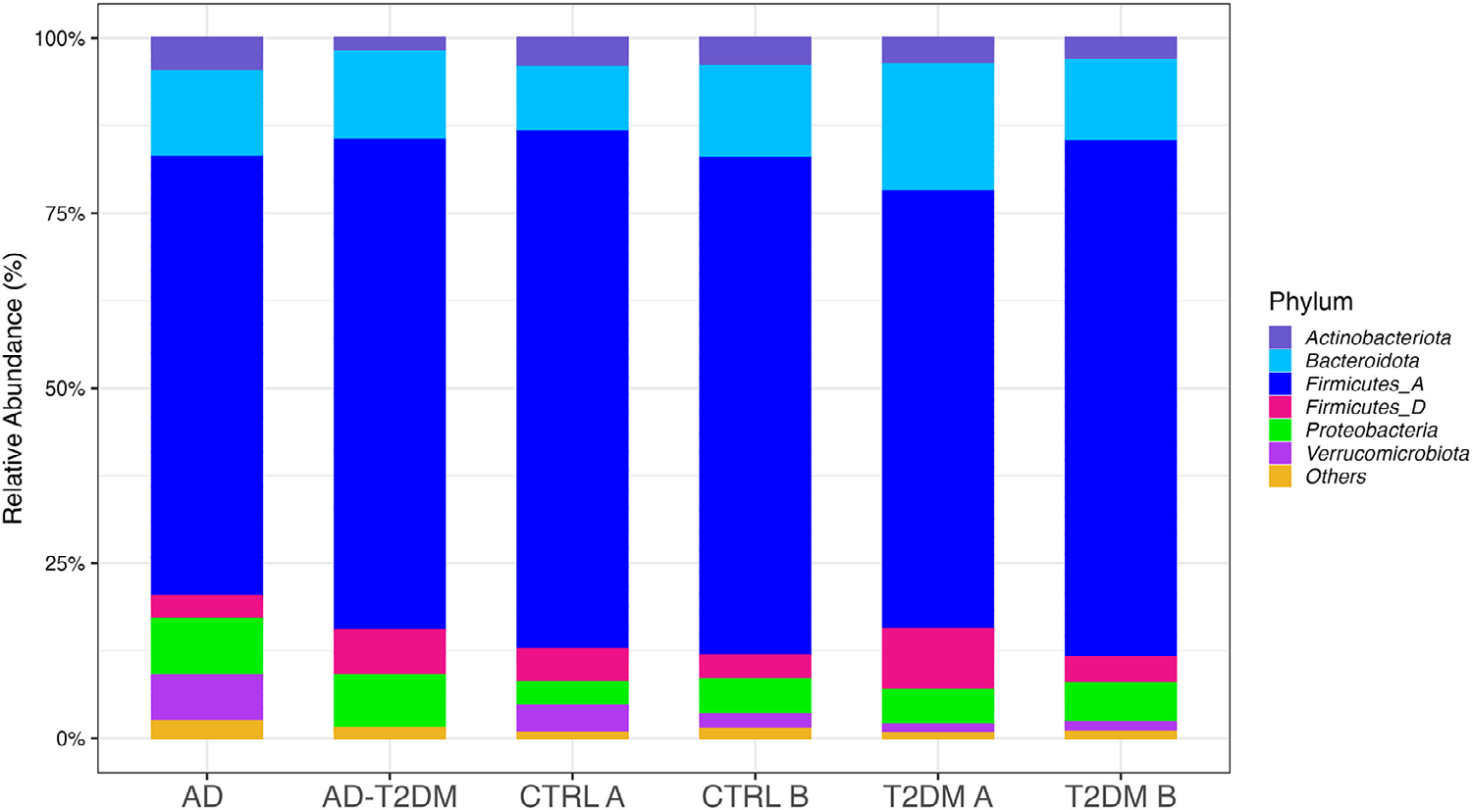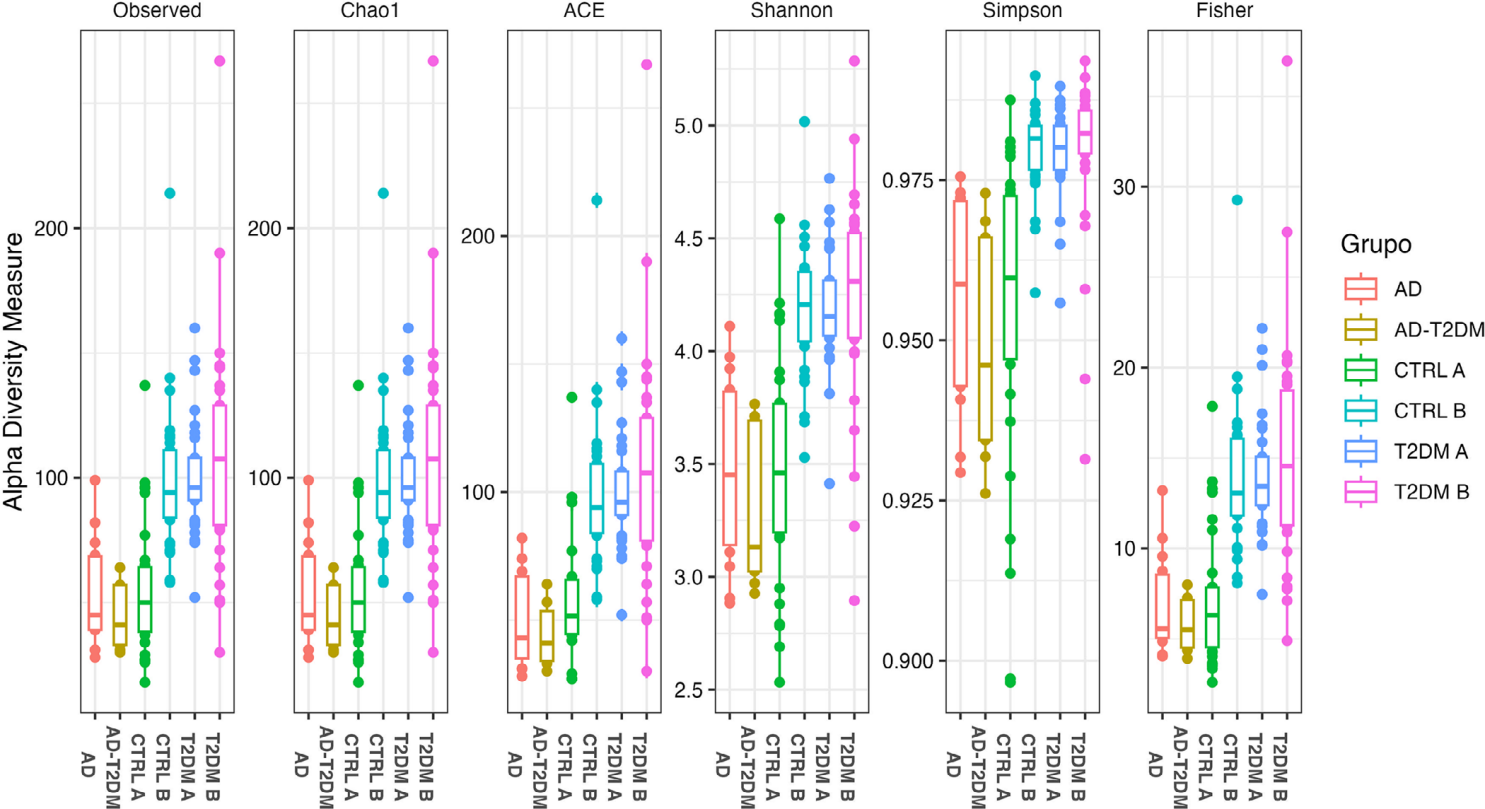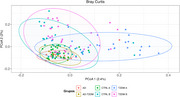# Characterization of the gut microbiota in type 2 diabetes mellitus‐Alzheimer's disease crosstalk

**DOI:** 10.1002/alz70855_097425

**Published:** 2025-12-23

**Authors:** Alexis M. Rodríguez Rosas, Diana L. Baldenebro, Carla E. Angulo Rojo, Alma M. Guadrón Llanos, Ángel Radamés Rábago Monzón, Jesus M. Perez Villarreal, Javier A. Magaña Gomez

**Affiliations:** ^1^ Universidad Autónoma de Sinaloa, Culiacan Rosales, SI, Mexico; ^2^ RORA9403301W0, Culiacan Rosales, SI, Mexico; ^3^ Universidad Autónoma de Sinaloa, Culiacán, SI, Mexico

## Abstract

**Background:**

Recently, the importance of the role of the gut microbiota in our organism has been highlighted, intervening mainly in immune activity and in the regulation of various metabolic pathways. Different studies have shown that both the gut microbiota of patients with T2DM and that of patients with AD present moderate dysbiosis, so we sought to characterize the gut microbiota of people with both pathologies.

**Method:**

The study population consisted of a total of 148 individuals, divided into four different groups: CTRL (*n* = 60), T2DM (*n* = 60), AD (*n* = 17), AD‐T2DM (*n* = 11). Stool samples were collected from the participants and DNA extraction was performed using the Quick‐DNA Fecal/Soil Microbe Miniprep Kit, following the manufacturer's instructions. Subsequently, the quality and concentration of the extracted DNA were evaluated by spectrophotometry using a NanoDrop 2000 (Thermo Scientific) and the integrity of the samples was verified by 1% agarose gel electrophoresis. The V4 region of the 16S rRNA gene was amplified by PCR. Library preparation was carried out using the Illumina MiSeq platform protocol. The raw sequencing reads were processed and phylum abundance analyses of the study groups were performed, as well as alpha and beta diversity to estimate differences within and between group samples.

**Result:**

The phylum *Verrucomicrobiota* showed significant differences in the AD group compared to the rest of the groups. In the case of the AD‐T2DM group, the phylum *Bacteroidota* was found to be significantly reduced when compared to the rest of the groups. Significant differences were found in the abundance of *Firmicutes_A* in the T2DM A subgroup compared to CTRL A. Regarding beta diversity, the CTRL A and CTRL B groups had a similar behavior, showing significant differences compared to the T2DM A and T2DM B groups.

**Conclusion:**

Gut microbiota dysbiosis in T2DM increases concomitantly with aging to a diversity similar to the gut microbiota of people with AD, contributing to the dysregulation of molecular networks leading to AD pathophysiology.